# A left atrial appendage thrombus that developed during prophylactic low‐dose dabigatran treatment resolved after switching to apixaban

**DOI:** 10.1002/ccr3.933

**Published:** 2017-03-31

**Authors:** Taku Koyama, Yoritaka Otsuka, Masaaki Kawahara, Yuki Imoto, Keita Nakamura, Sunao Kodama, Hiroo Noguchi

**Affiliations:** ^1^ Department of CardiologyFukuoka Wajiro HospitalFukuokaJapan; ^2^ Division of CardiologySugi HospitalFukuokaJapan

**Keywords:** Anticoagulants, atrial fibrillation, left atrial appendage, thrombus

## Abstract

We describe a case of atrial fibrillation in which an intracardiac thrombus that could not be prevented with “low‐dose” dabigatran treatment was resolved by switching to apixaban treatment. Thrombolysis using direct oral anticoagulants (DOACs) could be a therapeutic option for patients with intracardiac thrombi, although the efficacies of different DOACs seem to differ and need further examination.

## Introduction

Left atrial appendage (LAA) or left atrium (LA) thrombi are usually associated with atrial fibrillation (AF) and cause systemic thromboembolism complications. Therefore, patients with AF, particularly those at a high risk of thromboembolism, should receive anticoagulant therapy. Transesophageal echocardiography (TEE) is useful for the detection of LAA thrombi.

Compared to placebo or anti‐platelet therapy, warfarin is more effective in preventing thromboembolism complications in patients with AF 1, 2, 3. Recently, direct oral anticoagulants (DOACs) have been introduced as alternative prophylactics for thromboembolism in patients with nonvalvular AF. DOACs are associated with reduced risk of both stroke and bleeding compared to the standard warfarin 4, 5, 6. However, the differences in the efficacy of various DOACs for preventing intracardiac thrombi have not been studied, although some case reports have shown resolution of LAA thrombi with DOACs 7, 8, 9, 10. Here, we describe a case in which a LAA thrombus was resolved by replacing “low‐dose” dabigatran with apixaban.

## Case Report

**Figure 1:**
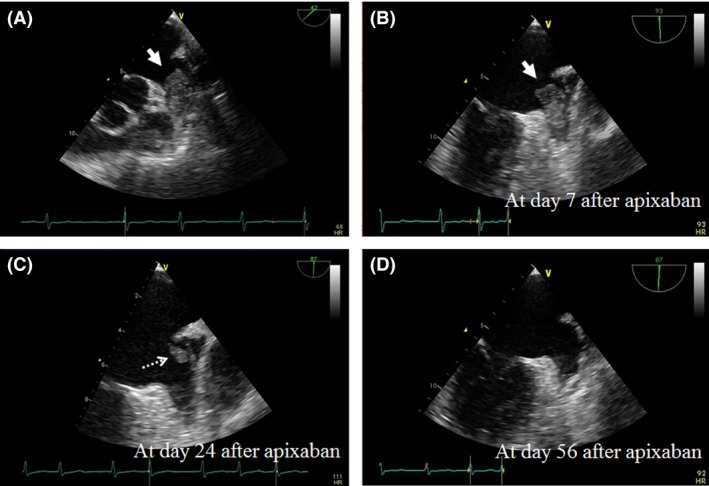
Transesophageal echocardiogram (TEE). (A) Left atrial appendage (LAA) thrombus (white arrow) was observed at first presentation. (B) No change of thrombus of LAA on day 7 after apixaban treatment. (C) Reduction in thrombus of LAA (white dotted arrow) on day 24 after apixaban treatment. (D) Complete resolution of LAA thrombus was achieved on day 56 after apixaban treatment.

A 78‐year‐old man with a history of hypertension was admitted to our hospital for aphasia, dysarthria, and numbness of the left leg. The patient initially received anticoagulant therapy with warfarin, but it was switched to 220 mg/day dabigatran by a private practice doctor over a year ago. A 12‐lead electrocardiogram at admission showed AF along with ST depression and negative T wave in leads V4–V6 and left ventricular hypertrophy. The CHADS_2_ and CHA_2_DS_2_‐VASc scores before cerebral infarction development were 2 and 3, respectively. Transthoracic echocardiography showed normal left ventricular wall motion and was unable to detect an intracardiac thrombus. Brain diffusion‐weighted magnetic resonance imaging on admission showed hyperintense areas in the frontal lobes on both sides, but magnetic resonance angiography showed no stenosis or occlusion in the cerebral arteries. The patient was diagnosed with acute cerebral infarction due to cardiogenic embolism. On day 11 after admission, TEE was performed after treatment of the acute phase of cerebral infarction and showed a large LAA thrombus (16 × 26 mm; Fig. 1A). The patient's weight was 63 kg; serum creatinine level, 0.81 mg/dL (normal range, 0.80–1.20); estimated creatinine clearance, 66 mL/min; and estimated glomerular filtration rate, 69.7 mL/min/1.73 m^2^. We decided to switch from dabigatran to other DOACs. The patient was eligible for standard‐dose apixaban therapy and did not fulfill any dose reduction criteria. Therefore, 220 mg/day dabigatran was switched to 10 mg/day apixaban on day 15 after admission. The patient was carefully monitored using serial TEE and physical examinations. TEE conducted on day 7 after initiation of apixaban treatment showed no change in the size of the LAA thrombus (Fig. 1B), but that conducted on day 24 did (Fig. 1C); further, TEE conducted on day 56 after initiation of apixaban treatment showed complete resolution of the thrombus (Fig. 1D). The patient had no recurrent stroke and is currently alive without any complications.

## Discussion

Numerous reports have described the resolution of LAA thrombi in patients treated with oral warfarin 1, 2, 3. Contrastingly, it has been reported that warfarin is not very effective in resolving large intracardiac thrombi, and LAA thrombi persist in 44% of patients treated with this drug 11. In addition, patients with persistent LAA thrombi have a poor prognosis 11. Therefore, optimal anticoagulant management is critical in patients with LAA thrombi.

DOACs are superior to warfarin in preventing stroke or systemic embolism in patients with AF and are associated with less bleeding and lower mortality 4, 5, 6. In contrast to the indirect action of warfarin, DOACs directly inhibit thrombin or factor Xa in the coagulant cascade. The inhibition of thrombin prevents its binding to fibrin and fibrin degradation products, whereby DOACs have thrombolytic properties 12. Inhibition of factor Xa blocks the generation of thrombin. Thus, DOACs have the potential not only to prevent de novo thrombi but also to resolve established thrombi. In fact, some case reports have confirmed resolution of LAA or LA thrombi with DOACs 7, 8, 9, 10. In two studies, rivaroxaban 7 and apixaban 10 were used as the first choice of anticoagulant therapy, and two others reported that dabigatran 9 and rivaroxaban 8 were used as the second choice of treatment after failure of warfarin. To the best of our knowledge, this is the first documented case report of LAA thrombus resolution with a switch to apixaban after failed “low‐dose” dabigatran treatment.

It is important to consider why dabigatran failed to prevent thrombus development, but apixaban effectively dissolved it the thrombus in the present case. First, the mean plasma trough concentration of dabigatran shows a fivefold range of variation 13, 14. In other words, the range of plasma concentrations of dabigatran varies widely among individuals. Second, a low dose of dabigatran may not have been adequate to prevent and resolve the LAA thrombus in our patient, unlike the standard dose of apixaban. Third, direct factor Xa inhibitors and direct thrombin inhibitors may have different in vivo effects on thrombolysis. Recently, it has been reported that direct factor Xa inhibitors inhibit thrombin generation and platelet aggregation derived via the tissue factor pathway to a greater extent than direct thrombin inhibitors do 15. Switching to other DOACs may be an alternative option if a DOAC fails to prevent and resolve LAA thrombi. However, these findings need to be confirmed through more large‐scale studies.

In conclusion, we described a case of AF in which an LAA thrombus that could not be prevented with “low‐dose” dabigatran treatment was resolved by switching to apixaban treatment. Thrombolysis using DOACs could be a therapeutic option for patients with intracardiac thrombi, although the efficacies of different DOACs seem to differ and need further examination.

## Authorship

TK: drafted the manuscript and contributed to treating the patient. YO: contributed to treating the patient and critically revised and edited the manuscript. MK, YI, KN, SK, and HN: contributed to treating the patient. All authors have read and approved the final manuscript.

## Conflict of Interest

There is no real or potential conflict of interest for any author. No author has relationships relevant to the contents of this manuscript to disclose.
